# Understanding the organisational influences on the quality of and access to primary care in English prisons: a qualitative interview study

**DOI:** 10.3399/BJGP.2023.0040

**Published:** 2023-09-05

**Authors:** Laura Sheard, Sue Bellass, Kate McLintock, Robbie Foy, Krysia Canvin

**Affiliations:** Health Sciences, University of York, York.; Manchester Metropolitan University, Manchester, and University of Leeds, Leeds.; University of Leeds, Leeds.; University of Leeds, Leeds.; Keele University, Keele, and University of Leeds, Leeds.

**Keywords:** health care access, health care professionals, organisations, patient perspective, prisons, primary health care, qualitative research, quality of care

## Abstract

**Background:**

Primary care for routine healthcare conditions is delivered to thousands of people in the English prison estate every day but the prison environment presents unique challenges to the provision of high-quality health care. Little research has focused on the organisational factors that affect quality of and access to prison health care.

**Aim:**

To understand key influences on the quality of primary care in prisons.

**Design and setting:**

This was a qualitative interview study across the North of England from 2019 to 2021.

**Method:**

Interviews were undertaken with 43 participants: 21 prison leavers and 22 prison healthcare professionals. Reflexive thematic analysis was undertaken.

**Results:**

The overarching organisational issue influencing quality and access was that of chronic understaffing coupled with a workforce in flux and dependence on locum staff. This applied across different prisons, roles, and grades of staff, and was vocally discussed by both patient and staff participants. Intricately related to understaffing (and fuelled by it) was the propensity for a reactive and sometimes crisis-led service to develop that was characterised by continual firefighting. A persistent problem exacerbated by the above issues was unreliable communication about healthcare matters within some prisons, creating frustration. Positive commentary focused on the characteristics and actions of individual healthcare professionals.

**Conclusion:**

This study highlights understaffing and its consequences as the most significant threat to the quality of and access to prison primary care. Strategies to address health care affecting prison populations urgently need to consider staffing. This issue should receive high-profile and mainstream attention to address health inequalities.

## INTRODUCTION

Primary care is delivered across a diverse range of settings in England including prisons, young offender institutions, secure mental health facilities, and immigration detention centres. Almost 80 000 people currently live in prisons across England and Wales^1^ but high rates of reimprisonment lead to a throughput of 250 000 contacts with the prison service per year.^2^ People in prison are more likely to have mental health or substance use problems, cognitive disability, communicable diseases, and long-term conditions than people who have never been incarcerated.^3^ Delivery of high-quality health care in prisons is important in order to reduce health inequalities and to affirm society’s commitment to social justice.^4^

The healthcare system in England, both inside and outside the prison gates, is under extreme pressure. However, the prison environment presents unique and difficult challenges for the delivery of care such as overcrowding, security concerns, a lack of trained staff, difficult staff recruitment and retention, and an archaic environment to practise in.^5^ Two-thirds of prison nurses who took part in a 2018 survey said the care they provided on their last shift was compromised and that the quality of care was poor.^6^ A recent analysis of prison inspection reports in Scotland found that a lack of healthcare staff meant that *‘the demand on existing staff to deliver a comprehensive range of services was almost at its ceiling’*.^7^

A qualitative study found that long waits for health care were a knock-on effect of a lack of custodial staff who did not have capacity to unlock people from their cells in time for healthcare appointments.^8^ Further, security concerns can disrupt access to treatment for people in prison and there is no equivalent comparison with health care delivered in the community.^9^

There is little research that focuses on the organisational factors influencing primary care in prisons and even less literature that pays attention to how these factors have an impact on quality and access. A notable exception are two papers by Ismail^8^^,^^10^ that concentrate on macroeconomic conditions, governance structures, and the impact of austerity. Further, most prison healthcare studies tend to prioritise discrete areas of health such as communicable diseases, mental health, or drug treatment services to the exclusion of routine healthcare conditions encountered every day in primary care such as asthma, hypertension, or diabetes.

The aim of this qualitative study was to understand what influences the quality of routine primary care in prisons alongside identifying the gaps and variations in care. In this study, the views of prison leavers and healthcare staff are explored regarding the main organisational drivers that influence the quality of prison primary care and access to it. The term ‘organisational’ is used to refer to the culture within or across prisons and the configuration of services and resources.

**Table 1:** How this fits in

Primary care services are delivered across many prisons every day in England but there is little previous research exploring the key organisational factors influencing quality of care and access in this setting. This qualitative interview study with people who had been in prison and prison healthcare staff found that understaffing—which then often led to a reactive and crisis-led service — was the core organisational issue that influenced quality and access. Understaffing is rife across many sectors of health care in England but it is particularly fraught within the prison estate where it collides with a higher disease burden and exacerbates health inequalities. Factors that influence the quality of and access to prison health care deserve to receive mainstream research and policy attention.

## METHOD

A qualitative interview study was conducted with people who had previously lived in prison and prison healthcare staff across the North of England.

### Sampling and recruitment

Purposive sampling was undertaken to ensure participant diversity. For the people who had lived in prison, this related to age, gender, ethnicity, and health condition. For prison healthcare staff, this related to security categories of prisons they had worked in and length of professional service.

The approach to recruitment of people who had lived in prison included: approaching local service providers, personal networks of personal and public involvement consultants, Twitter adverts, and snowballing. Prison healthcare staff were invited to take part through an email circulated among staff at two third-sector providers of prison health care, via Twitter, and snowballing. Inclusion criteria for the study were: experience of receiving or delivering any type of primary care in any prison during the previous 3 years before the interview date.

### Participants

Data collection took place between November 2019 and March 2021. Interviews were conducted with 21 people who had received health care while in prison and 22 people who had delivered prison healthcare in a professional role (totalling 43 participants).

The prison leavers sample consisted of 17 males and four females, which reflects the English prison population being overwhelmingly male. The age range was 27–60 years. Participants identified as the following ethnicities: 14 were White, three were Asian, two were Black, one was of mixed ethnicity, and one chose not to disclose. Health conditions included not only substance misuse and mental health issues but also long-term physical conditions such as diabetes, lupus, and HIV. Participants had resided in a wide range of prisons (*n* = 48) across England including open and closed prisons, and the full range of male security categories (A to D). Around halfway through recruitment, the authors of this study looked in detail at the sample to understand demographics or experience that were not represented. As it was found that there was not sufficient representation of females or people with long-term chronic health conditions, the authors’ attention turned to specifically recruiting participants with these characteristics.

The prison healthcare staff sample consisted of 16 males and six females, with an age range of 26–62 years and all being White British apart from one. Length of service ranged from <1 year to over 12 years. Staff participants had experience of working across both the male and female estate and all security categories of prison. Staff roles included: junior and senior nurses including a nurse prescriber, GPs, heads of health care, occupational therapists, recovery workers, a physiotherapist, an associate practitioner, and a pharmacy technician.

### Interview conduct

A researcher (the second author) with a background in social science and expertise in qualitative research and sensitive interviewing techniques undertook all the data collection. Interviews with former patients were conducted after people had left prison, mostly via phone. Interviews with healthcare staff were all conducted over phone or video call. Interviews with patients were on average 35 min long (range 18–73 min) and interviews with healthcare staff were on average 46 min long (range 31–61 min).

The authors chose to interview people who had left prison (by phone, video or face-to-face) rather than individuals who were currently residing in prison for several reasons:

- to build a comprehensive picture about quality and access across the North of England, therefore selecting certain prisons may have been too restrictive;
- people could participate at a time convenient for them rather than the day when the researcher would be visiting the prison; and
- less burden for prison establishments if researcher access/escort was not required.

A few months into the project the COVID-19 pandemic struck, hence the vast majority of interviews were conducted over the phone.

All interviews were conducted using a topic guide to ensure consistency across participants; however, a flexible format allowed participants to voice what they considered important. One topic guide was developed for each participant group, in consultation with people who had lived experience, clinicians, and engagement with the literature. The topic guides focused on the experiences of delivery or receipt of prison healthcare, barriers and facilitators to care, the journey through the prison healthcare system, access to primary and secondary care, comparisons with community primary care, and health data infrastructure.

### Analysis

All interviews were transcribed verbatim. There were two stages to the analytic process. First, all data were mapped onto a matrix depicting four levels of quality in health care:^11^

- individual;
- group/team;
- organisational; and
- system.

Perceptions of barriers and facilitators that impacted on high-quality prison health care were identified in the data for both patients and staff at each of the four levels, where possible. The findings of the data mapping at all levels was then written up descriptively by the second author.

Second, the research team noticed the importance of the organisational-level factors and undertook a closer examination. The first author conducted a reflexive thematic analysis^12^ that aims to identify patterns of shared meaning underpinned by a central concept. The first author returned to the original transcripts and undertook selective coding about organisational factors. Preliminary themes were constructed and sense checked with the other authors. The first author then conducted further interpretive work to write up the findings.

## RESULTS

**Figure 1.**
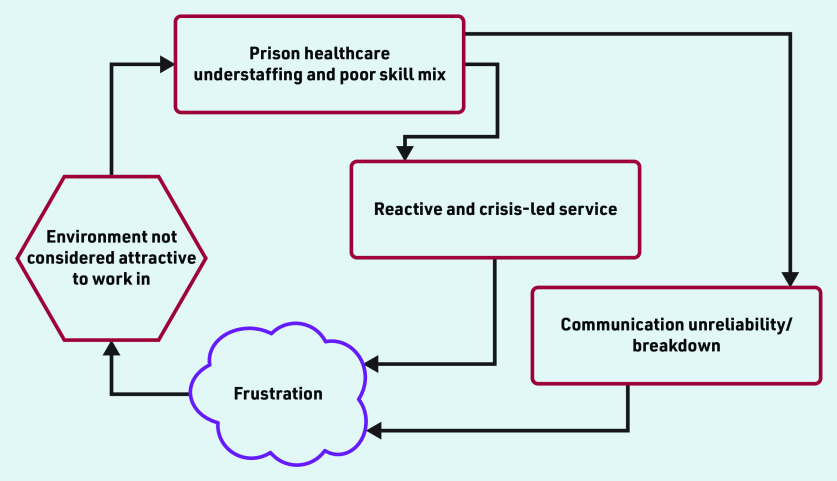
*Relationship between themes that affect the delivery of and access to high-quality prison health care.*

The study identified three main organisational level themes that were all intricately related to each other and demonstrably had a negative impact on the delivery of and access to high-quality prison health care. The themes are: (a) chronic understaffing, leading to, (b) a reactive and crisis-led service that is additionally hampered by, (c) continually disrupted communication, which hinders healthcare access (Figure 1). Positive elements of healthcare provision that came through in the interviews are also included.

### Chronic understaffing and a workforce in flux

The overarching issue affecting the quality of prison health care was chronic understaffing and a workforce in flux increasing dependency on the use of locum staff. Understaffing also applied to prison officers and this is explored further in the third theme. Both people in prison and healthcare staff explicitly commented on the lack of healthcare staff available to deliver core services. This occurred across different types of healthcare roles, grades, and, most importantly, across a variety of different prisons and security categories. Over half the staff participants talked pointedly about the persistent understaffing they regularly encountered:

*‘ We are short of staff. We haven’t got the staff at the minute so not only have you got* [nurse] *running her own wing … she is often running other clinics that didn’t have a nurse because there were no staff to staff it.’* (Healthcare professional [HCP] 7, allied health professional, female estate [these refer to female prisons that are either open or closed and not in categories from A–D, as is the case for male prisons])

*‘ The problems would be eradicated if the staffing levels were sufficient. It all comes down to too much work and not enough time and staff.’*

(HCP 17, mental health nurse, female estate)

People in prison were conscious of the scarcity of staff as a result of their healthcare interactions (which were reported as *‘rushed’*) and the experience of long waits to be assessed not only at the first night reception clinic but also mental health and nurse appointments, and excessive queues for the medication hatch.

People in prison often remarked that healthcare staff were doing the best they could while acknowledging that staff were working within a constrained system:

*‘Most of the time they are really rushed because they are understaffed. Big queue of lads waiting.’*

(Patient 8, 44 years, male [M])

*‘ They* [healthcare staff] *are proper overworked, they are, but for the amount of people that are in the jail to the amount of staff that are there, it’s pathetic.’*

(Patient 5, 41 years, M)

*‘ Ninety-nine per cent of the time I’ve had good experiences with them* [healthcare staff]*, it’s just actually getting something. But that’s not down to them, that’s down to the system they have to work to.’*

(Patient 4, 30 years, M)

Alongside the clear acknowledgement of staff shortages, some healthcare staff participants discussed the issue of a significant amount of the existing workforce comprising locums or agency staff. This was considered problematic, leading to a lack of a substantive permanent workforce who knew the prison and the patients within it, meaning that staff had minimal time, head-space, or continuity of service to undertake quality or service improvement work:

*‘ My vacancy rate is about fifty per cent of core staffing levels. I am having to backfill with agency.’*

(HCP 10, senior clinical manager, male category A prison)

*‘ It’s about … having clinicians who are interested in what they do and obviously with some of the locums you are not getting the buy-in that we do from your substantive staff, so not getting the people who are interested in creating new pathways, implementing new ideas.’*

(HCP 9, lead GP, works across multiple prisons: male, female, and categories)

Regarding the above, it is noteworthy that some participants discussed how there was an absence of a long-term approach to care planning and planning for future service provision. A few healthcare staff participants commented on the difficulties of attracting clinicians to work in the prison setting. This was attributed to the challenges of the environment and the complexity of the patient population, which was felt to be many times greater than what would ordinarily be expected in community primary care.

### A reactive and crisis-led service

A consequence of understaffing was the propensity for service provision in some prisons to tip into being reactive rather than planned and, in parts, crisis led. This had demonstrable effects on service provision. Healthcare staff participants talked about not being able to perform — or having to delay — routine but core aspects of their role as continual firefighting became the norm:

*‘ You’ve got very few nurses, sometimes you are having to look at “ Well what do I cancel first? ” and obviously priorities have to be things like medication, any emergencies, all the urgent stuff, so a lot of the times we were finding that the long-term condition clinics were the ones that were getting cancelled or getting put on hold or delayed.’*

(HCP 3, senior clinical manager, male category D prison)

*‘ Since I’ve worked here there’s been a big staff turnover, I’ve noticed. People don’t stay and I think that’s because of the difficult environment but also because every day it does seem like it’s firefighting.’*

(HCP 19, pharmacy technician, male category A prison)

A reactive service due to understaffing meant that patients trying to seek help for perceived minor problems were sometimes put off or ignored until the problem escalated and became a major issue that staff were then forced to deal with as their top priority on that shift. This applied to both physical and mental healthcare provision. Regarding physical health, there were examples of patients’ important medication about to imminently run out despite staff being repeatedly reminded about it (sometimes for weeks). However, mental health took the lion’s share of concerns from both staff and patient participants as minor problems could eventually lead to serious consequences. Patients spoke about how they knew others in the prison who had self-harmed because staff were only able to deal with high-priority patients, so self-harming was sometimes seen to be the only way to secure medical attention. Staff participants acknowledged the unmet demand for mental health professionals perceived as being papered over with the use of medication instead of talking therapies:

*‘ A lot of the things I experienced talking to some of the lads were, “ You have cut your arm* [self-harm]*, it is bandaged up, now get back to your cell.” It wasn’t, “ Why have you done that? ” It is just, “ When it happens, we will deal with it.” There is no preventing.’*

(Patient 10, M, 29 years)

*‘ There are a lot of people who really need somebody to talk to. They don’t need medication, they don’t need high-level mental health input, but they do need somebody to talk to, and because the mental health team are so busy, that comes to us, whereas really we’re not counsellors, or not very good counsellors … We end up giving them medication when really what they’d benefit from more is a course of counselling or more access to talking therapies.’*

(HCP 5, GP, male category C and D prisons)

The COVID-19 pandemic and its influence on prison life was discussed by many participants, although most of the prison leavers had been released before the start of the pandemic. Healthcare staff described COVID-19 as *‘a logistical nightmare’* that further stretched an already overloaded system and aggravated all of the issues explored above.

### Continual communication unreliability that hinders healthcare access

A further problem, exacerbated by understaffing, was that of communication processes related to access to healthcare being persistently unreliable. Interviewees from both groups described a lack of responsibility for effective communication on healthcare matters and excess pressure on overburdened officers and healthcare staff, resulting in communication with patients often slipping between the responsibilities of the two professional groups.

Participants repeatedly mentioned the fallible process of how patients requested a healthcare appointment and how a patient was informed about the outcome of their request. Briefly, this took the form of physical slips of paper in the form of an application (‘apps’), which had to pass from the patient as the sender through various offices before getting to the recipient (although in some prisons this process is computerised). The physical slips are often transported via prison officers or other third parties external to the healthcare department. The process was reversed for communication back to the patient:

*‘ … type a letter saying “ We’ve made you an appointment ”, not even knowing whether that letter will get to the prisoner because you send the letter down to the admin office, that goes to the wing office, then it’s supposed to go out to the prisoners, but quite often prisoners don’t seem to get the written communication that we send.’*

(HCP 5, GP, male category C and D prisons)

This lengthy and convoluted process was a persistent source of tension, with patients being informed that they had missed healthcare appointments that they were unaware had been made for them, sometimes after waiting weeks or even months to hear back. Some patients described how they never heard back about their appointment request despite submitting it multiple times, leading to frustration:

*‘You feel as if what was the point in telling you that I might need help with mental health, that I don’t feel really well or I’m losing loads of hair, or whatever it may be, what was the point in telling you when you weren’t going to do anything?’*

(Patient 17, female, 39 years)

Compounding this issue was the lack of available prison officers often leading to people in prison not being unlocked from their cell in time for a healthcare appointment. These factors all led to a demonstrably high rate of ‘did not attend’ for healthcare appointments that were often coded in patients’ medical notes without reference made to whether non-attendance was a system fault or down to the patient. Tension and frustration about all the above was created and maintained at multiple levels with prison officers, healthcare staff, and patients all becoming frustrated with each other:

*‘ Sometimes you might get it slipped through your door saying that you have an appointment on Tuesday and your door never gets opened up by an officer to go to your appointment.’*

(Patient 9, M, 39 years)

*‘ But sometimes it isn’t their* [patient’s] *fault but it can be coded as a “ failed to attend ” and that makes the figures look convenient. It’s easy to say they didn’t go, isn’t it? You know that and I know that.’*

(HCP 15, senior nurse, female estate)

The above related issues of communication unreliability and high rates of non-attendance demonstrate how the prison system itself can significantly impact on primary care delivery.

### The importance of relationships between people

In contrast with the above constraining themes, positive commentary tended to focus on characteristics and actions of individuals working within the service rather than elements of the system itself. Many people in prison found interactions between themselves and individual members of healthcare staff to be positive and talked about mostly being treated with respect by the clinician in front of them. Some clinicians talked passionately about how personally rewarding it was to work with people in prison:

*‘ I love working with prisoners, they are the most motivated, engaged, enthusiastic, grateful, everything that people wouldn’t think that they are. They are just the most endearing group of people … that I’ve ever worked with, so I stick with it because they are amazing. The rest of it is utterly hideous, to be honest.’*

(HCP 20, allied health professional, male category B)

*‘ The healthcare staff gave you their time, they listened to what you said, they asked you questions as if you were in the community. Specifically the HCAs* [healthcare assistants] *and nurses were attentive.’*

(Patient 11, M, 36 years)

Elements of good practice were highlighted by people who had lived in several different prisons and could compare healthcare services. Comparisons were often made about the difference in healthcare services between private and publicly run establishments but opinion varied as to which was better managed and staffed. Healthcare staff participants expressed a preference for working for specific named healthcare providers with higher expectations of care.

## DISCUSSION

### Summary

Chronic understaffing was the most significant influence on prison healthcare quality and access. This led to a reliance on a significant locum workforce. Understaffing nudged the service in many prisons to be reactive and crisis led with continual firefighting becoming the norm. Persistent communication unreliability, particularly about healthcare appointments, further exacerbated healthcare provision. Overall, the above identified interrelated themes could be described as a vicious cycle leading to a depleted workforce, which then creates an environment not considered attractive to work in. This in turn manifests itself as a difficulty in attracting a dedicated, talented, and permanent workforce resulting in further staff depletion (Figure 1). Positive opinion was expressed by both staff and patients about aspects of health care but this tended to focus on the relationships between people at an individual level.

### Strengths and limitations

The perspectives of both people who had been in prison and healthcare staff converged, which lends more confidence to the findings than if the main themes had been derived from only one participant group. The sample of prison leavers was diverse with representation from ethnic minority groups, females, and those living with long-term health conditions. The authors did not place restrictions on which establishments people had lived or worked in, leading to participants from the male and female estate and all security categories. In addition, the authors did not narrow the focus to specific health conditions, thereby leading to a broad understanding of the influences on healthcare delivery.

A limitation of the study is that interviews were conducted with people following their release from prison, which could potentially have led to recall bias. Despite the wide range of participant experience, interviews were limited to those who spoke English. The study took place predominantly across the North of England so the findings may not be reflective of prison healthcare systems in other countries including Wales, Scotland, and Northern Ireland.

### Comparison with existing literature

The main factors identified in this paper are in keeping with several other studies that have considered understaffing as an issue for delivery of primary care in prisons, but usually only one issue among many.^7^^,^^13^^–^15 Understaffing of prison officers has been noted previously as detrimental to healthcare access and contributing to increased waiting time.^8^

A qualitative study with prison policymakers highlighted a dilution of both officer and healthcare staff workforce that happened as a result of austerity and a deteriorating and distracted governance structure.^10^ Other literature from England has looked at access to and quality of health care in terms of relationships and interpersonal communication between patients and GPs.^16^ The security constraints in access to secondary care for people in prison has been examined^9^ and quantitative research has found that 40% of hospital outpatient appointments made for people in prison are not attended.^17^ All of the above is compounded by prison healthcare staff working in what has been termed ‘an entrenched closed culture’ where a disconnect is said to exist not only between the prison and NHS systems but also between health care and custodial staff within the same prison.^18^

Recent literature has demonstrated that COVID-19 had a significant impact on the provision of health care, especially in relation to staff shortages.^19^ A survey study from the US found the main challenges to prison clinicians’ roles were constraints around being able to deliver care effectively and safely.^15^ This was mainly attributed to ineffective leadership alongside understaffing and lack of resources.

The current study posits understaffing and its knock-on effects as the major issue influencing quality and access based on the perceptions of both patients and healthcare staff across England prisons.

### Implications for research and practice

The fundamental foundation of every healthcare service is its workforce but the current findings have demonstrated that the prison healthcare workforce is severely depleted. Prison healthcare provision in the UK is provided by a fragmentation of third-sector and for-profit companies, with healthcare staff employees often receiving terms and conditions that are viewed as inferior to those of their peers in the community^10^ coupled with a lack of career development. Prison healthcare careers should be made more attractive with clear career trajectories, better terms and conditions, protected time for training, and investment in upskilling of current staff. This is only likely to come to fruition when the time period for procurement cycles of competitive tenders are significantly extended to provide continuity of service and discourage a race to the bottom.

Understaffing is rife across most sectors of health care currently in the UK. Community primary care is struggling to meet the demand from a growing and ageing population, with a national GP vacancy rate of 17%^20^ and all healthcare sectors have been disadvantaged by a high turnover of nursing staff leaving the service because of stress and burnout.^21^ Additionally, mental health care delivered in the community suffers from a vicious cycle of chronic understaffing.^22^

The most pertinent issues raised in this paper such as poor staffing levels and communication problems are just as likely to be found in ‘deep end’ (most deprived)^23^ GP practices in the community. Therefore, it is not a novel or surprising finding that understaffing also applies to prison health care and is likely to be one of the main factors hindering quality and access. Rather, it is anticipated that the current findings will inherently resonate with both those working in prison primary care and those who have been in prison, therefore the findings can be used to explicate, highlight, and escalate their lived experience of both delivering and receiving health care under challenging conditions. Related to this, there has been a recent emphasis placed on addressing health inequalities and understanding the wider determinants of health^24^^,^^25^ within the policy and research discourse in the UK.

A key enabler of tackling health inequalities is to understand how they are enacted and who they disproportionately affect. In order to do this, factors influencing provision of prison healthcare deserve to receive mainstream research and policy attention, on a par with the attention directed to what is inhibiting healthcare delivery in the community and acute sector.
